# Product Distribution of Steady–State and Pulsed Electrochemical Regeneration of 1,4‐NADH and Integration with Enzymatic Reaction

**DOI:** 10.1002/open.202400064

**Published:** 2024-04-12

**Authors:** Mohammed Ali Saif Al‐Shaibani, Thaleia Sakoleva, Luka A. Živković, Harry P. Austin, Mark Dörr, Liane Hilfert, Edgar Haak, Uwe T. Bornscheuer, Tanja Vidaković‐Koch

**Affiliations:** ^1^ Electrochemical Energy Conversion Max Planck Institute for Dynamics of Complex Technical Systems Sandtorstraße 1 39106 Magdeburg Germany; ^2^ Institute of Biochemistry University of Greifswald Felix-Hausdorff-Str. 4 17487 Greifswald Germany; ^3^ Institute of Chemistry Otto von Guericke University Universitätsplatz 2 39106 Magdeburg Germany

**Keywords:** cofactors, direct electrochemical 1,4-NADH synthesis, dynamic operation, enoate reductase, chemoselectivity

## Abstract

The direct electrochemical reduction of nicotinamide adenine dinucleotide (NAD^+^) results in various products, complicating the regeneration of the crucial 1,4‐NADH cofactor for enzymatic reactions. Previous research primarily focused on steady–state polarization to examine potential impacts on product selectivity. However, this study explores the influence of dynamic conditions on the selectivity of NAD^+^ reduction products by comparing two dynamic profiles with steady‐state conditions. Our findings reveal that the main products, including 1,4‐NADH, several dimers, and ADP‐ribose, remained consistent across all conditions. A minor by–product, 1,6‐NADH, was also identified. The product distribution varied depending on the experimental conditions (steady state vs. dynamic) and the concentration of NAD^+^, with higher concentrations and overpotentials promoting dimerization. The optimal yield of 1,4‐NADH was achieved under steady–state conditions with low overpotential and NAD^+^ concentrations. While dynamic conditions enhanced the 1,4‐NADH yield at shorter reaction times, they also resulted in a significant amount of unidentified products. Furthermore, this study assessed the potential of using pulsed electrochemical regeneration of 1,4‐NADH with enoate reductase (XenB) for cyclohexenone reduction.

## Graphical abstract

This study investigates the direct electrochemical reduction of NAD^+^ for enzymatic cofactor regeneration. It discovers that dynamic conditions improve the short time selectivity towards 1,4‐NADH, but also result in the production of inactive by–products. The highest yield is achieved under steady–state conditions, marked by minimal overpotential and prolonged operation time. While the reduction of cyclohexenone using XenB in conjunction with the direct electrochemical reduction of NAD^+^ is possible, practical limitations exist.

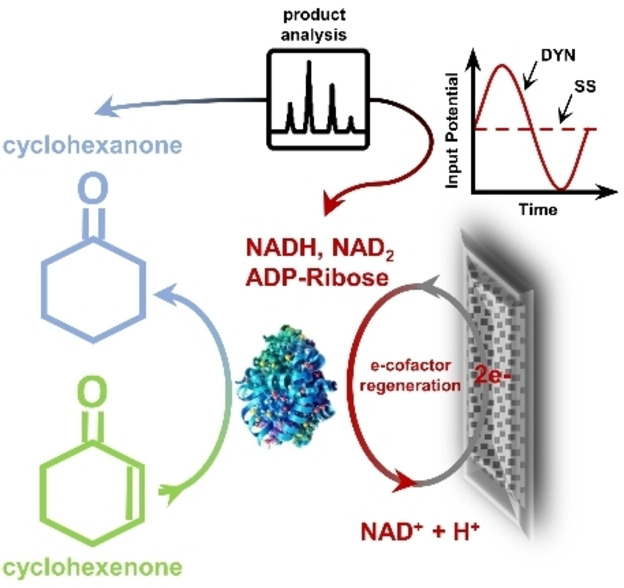

## Introduction

Oxidoreductases are an important class of enzymes^[1]^ that catalyze oxidative or reductive biotransformations, more specifically they transfer electron or redox equivalents, from a donor molecule to the corresponding acceptor molecule.^[2]^ The enzymatically catalyzed redox reactions have thus been the focus of scientific interest due to their potential in a variety of industrial applications.^[3]^ Despite this, they have not yet been widely used on an industrial scale due to limitations common to this class of enzymes, such as low stability and cofactor dependency.^[4]^ Specifically, they require a hydrogen source, i. e., a cofactor, to act as a shuttle that transfers the charges between the substrate and the enzyme.^[5]^ The best examples of these cofactors are nicotinamide adenine dinucleotide (NADH) and its phosphorylated form nicotinamide adenine dinucleotide phosphate (NADPH), which act as electron donors for 90 % of the known oxidoreductases,[^3b^, ^6^] with the other 10 % comprising of enzymes using flavin,^[7]^ PQQ^[8]^ or other cofactors. Due to the high cost of the cofactors, their use in stoichiometric quantities is uneconomical on an industrial scale.^[9]^ Therefore, an efficient cofactor regeneration method is required. Currently, enzymatic cofactor regeneration is the most utilized.^[10]^ This involves the use of secondary enzymes such as glucose‐ or formate‐dehydrogenases (GDHs or FDHs) as well as alcohol dehydrogenases (ADHs) to regenerate the NADH or NADPH consumed during the reaction of interest.^[11]^ This has some disadvantages, such as pH changes and the formation of a large amount of byproducts (196 g of gluconic acid is formed per mole of NADH),^[12]^ causing negative effects on enzyme stability and activity as well as high separation costs.

To overcome these limitations several alternative approaches for cofactor regeneration have been suggested such as electrochemical, chemical, and photochemical.^[13]^ All of these have distinct advantages and disadvantages, and currently, no cofactor regeneration method is fully accepted. Electrochemical regeneration of cofactors employs (renewable) electricity instead of a sacrificial carbon‐based substrate, which has the general advantage of improving the CO_2_ balance, thereby avoiding the formation of further byproducts derived from these compounds. Furthermore, electrochemical processes have very low E‐factors (kg waste/kg product) compared to other regeneration strategies.^[13a]^ Especially promising are direct cofactor regeneration electrochemical strategies. They ideally require only electrons and protons to regenerate NAD(P)H cofactors, in theory forming no waste products. In practice, however, this is not always the case. This is especially evident when unmodified electrodes are used in NAD^+^ reduction (NADRR), which often leads to undesired product formation and in practice often exhibits low selectivity in forming enzymatically active 1,4‐NADH. However, the selectivity towards 1,4‐NADH varies between different electrode materials. It was shown that the formation of 1,4‐NADH on bare Au, bare Cu, and Pt‐modified Au (Pt−Au) electrodes at the cathodic potential of −1.057 V vs. Ag/AgCl was 30 %, 52 %, and 63 % respectively. Very high yields of 1,4‐NADH were reported on a glassy carbon electrode (98 %) at −1.797 V vs. Ag/AgCl and on a bare Ti electrode (96 %) at (−0.997 V vs. Ag/AgCl).^[14]^ An additional consideration to the choice of electrode material, is the potential, a factor which largely determines product selectivity.^[15]^ This was demonstrated by Ali et al.^[13g]^ who found that on a glassy carbon electrode at a low cathodic potential (−0.957 V vs. Ag/AgCl), 32 % of the converted NAD^+^ resulted in 1,4‐NADH, whereas, at a high cathodic potential, this amount reached 98 %.

The selectivity issue is not inherent to NADRR, but it is a general problem in electrosynthetic applications,^[16]^ as was shown recently, in CO_2_ reduction reaction (CO2RR) experiments.^[16b]^ In this context, dynamic operation i. e., pulsed electrolysis is lately been discussed as a promising approach to tuning CO2RR selectivity towards certain products. Most of these studies employed rectangular wave signal potential pulsing between cathodic and anodic regions.^[17]^ Under such conditions, the catalyst was constrained to reduction–oxidation cycles. Therefore, the observed selectivity improvements were mainly rationalized in terms of catalyst changes. However, also studies where the current sign was not changing and the current was pulsed, between less and more negative current values, reported changes in the product selectivity. In this case, no changes in the catalyst surface were observed. In an effort to provide a theoretical explanation for these observations, our group recently suggested a so–called direct current (DC) contribution as a cause of selectivity improvement under dynamic conditions.^[18]^ DC‐contribution is a non–periodic part of the non–linear frequency response.^[19]^ It can be considered a quantitative measure of process improvement or process deterioration under dynamic conditions. The origin of this effect is in the nonlinearities of electrochemical processes, with nonlinear electrochemical kinetics mainly contributing. We have shown that the selectivity of CO with respect to hydrogen can be tuned by selecting proper forcing parameters (e. g. amplitude, frequency, etc).^[18]^

In this study, we examine the impact of dynamic operation on the product distribution of NADRR. For comparison, we investigate the product distribution of NADRR under steady–state conditions as a reference case. Our focus is on carbon–based electrodes, chosen due to carbon‘s low cost and potential for enhanced selectivity tuning. Furthermore, carbon‘s frequent use in other methods, such as mediated electron transfer NADRR, underscores its relevance.[^13d^, ^20^] With respect to direct electrochemical NADRR, in addition to the already mentioned study by Ali et al.^[13g]^ on glassy carbon, the same group reported on NADRR on high surface area carbon materials.^[21]^ The conclusions from both studies were similar. It was communicated that an increase of overpotential is increasing product selectivity towards 1,4‐NADH. The conversion towards 1,4‐NADH form was based on spectrophotometric readings at 340 nm and an enzymatic assay. Other products were not quantified. Contrary to these observations, in a recent paper by Liu et al.^[22]^ an increase of dimer formation with an increase of an overpotential followed by a decrease of active 1,4‐NADH isomer on carbon felt electrode was observed. In that study product distribution of NADRR was quantified with the help of NMR measurements and enzymatic assay/UV‐VIS spectrometry. The approach relied on NMR spectroscopy for the qualitative identification of products and determination of product concentration ratios (e. g. between 1,4‐NADH and 1,6‐NADH). It was shown that the main products of NADRR on carbon–based electrodes are ADP‐Ribose and dimers. The dimers were identified based on ^1^H‐NMR signals, but also electrospray ionization mass spectrometry (ESI‐MS).

In all literature studies on NADRR on carbon materials quantitative product distributions were based on the use of enzymatic assays (e. g. lactate dehydrogenase (LDH)^[15]^ or lipoamide dehydrogenase)^[21a]^ and UV‐VIS spectrometry readings at 340 nm. The drawback of this approach might be enzyme inhibition by 1,6‐ and 1,2‐NADH isomers.^[23]^ In this study, the distribution of products was analyzed using high–performance liquid chromatography (HPLC) equipped with a UV‐VIS detector, and gas phase product distribution was assessed using online gas chromatography (GC). The impact of various conditions on the distribution of NADRR products, including NAD^+^ concentration, electrical potential, flow rate, and pH, was investigated. Comparisons were made between product distribution under stationary polarization and dynamic operation. The potential of dynamic operation to enhance the selectivity of unmodified electrodes for the enzymatically active 1,4‐NADH isomer was evaluated. As a proof of concept, we integrated direct electrochemical cofactor regeneration under dynamic conditions with an enzymatic reaction–a novel approach for carbon–based porous electrodes. This configuration enabled the transformation of cyclohexenone into cyclohexanone, catalyzed by enoate reductase, albeit with certain limitations.

## Results and Discussion

**Figure 1:**
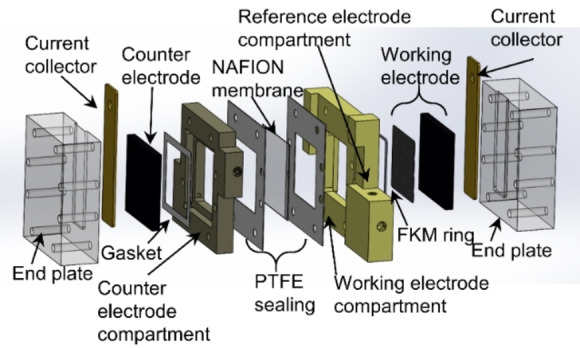
Exploded view of a flow reactor setup with (from left to right) end plate, current collector, glassy carbon counter electrode (CE), the CE compartment, PTFE sealings, NAFION membrane separating the CE and working electrode (WE) compartments with an integrated reference electrode compartment, sealing ring, WE, WE current collector, and end plate.

Direct electrochemical NADRR investigations were conducted using various carbon–based electrodes, including a rotating disk GC electrode, a GC electrode within a flow cell setup, and a porous electrode made of carbon nanoparticles also situated in a flow cell setup (Figure 1).

**Figure 2:**
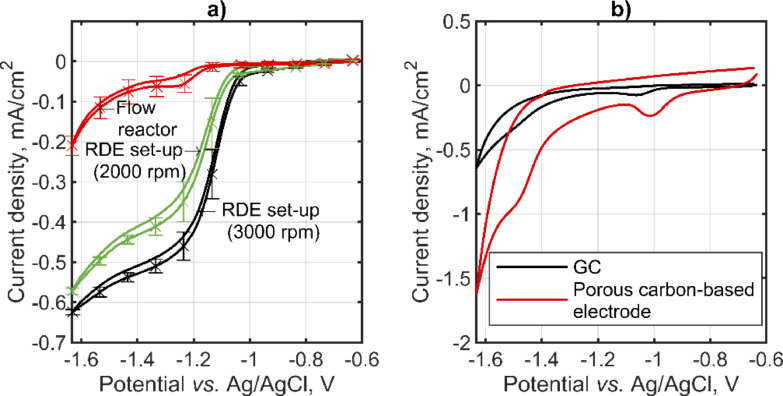
Electrochemical NADRR on different carbon–based electrodes in a) RDE set up at two different rotation rates and flat GC electrode in a flow cell setup, and b) flat GC and porous carbon nanoparticle electrode on Toray paper support featuring 0.88 mg cm^−2^ carbon nanoparticle loading and 2.5 wt % PTFE in a flow cell setup. Conditions: Figure 2 a) 20 mV/s scan rate, 1 mM starting NAD^+^ concentration, 0.05 sodium phosphate buffer (pH 7.5), 22 °C flow rate 80 ml/min, and rotation rates of RDE as indicated. Conditions: Figure 2 b): 20 mV/s scan rate, 2 mM starting NAD^+^ concentration, 0.1 M sodium phosphate buffer (pH 7.5), 22 °C and stagnant conditions. The reference electrode was RHE, but all potentials were recalculated with respect to Ag/AgCl.

Typically, the electrochemical NADRR process on carbon‐based electrodes is marked by the emergence of a limiting current. This limiting current is influenced by mass transport, as evidenced by the fact that an increase in rotation rate (illustrated in Figure 2a) leads to an elevated limiting current. Such findings are consistent with observations reported in the literature.^[13g]^ If instead of a rotating disc electrode (RDE), a GC electrode in the flow setup has been used, a significant decrease of the limiting current has been observed as well as a shift of an apparent onset potential to a more negative value. This is likely due to different flow conditions in two setups, as already observed in our lab on an example of CO2RR, indicating not–so–optimal flow conditions in the flow cell. At potentials more negative than −1.4 V vs . Ag/AgCl, an onset of hydrogen evolution reaction can be observed in all tested systems. With this, there is a potential window in which NADRR is not significantly influenced by hydrogen evolution reaction (HER) (see hydrogen Faradaic efficiency, Figure S1). The existence of a potential window where NADRR is not accompanied by HER makes carbon–based materials different than other metal–based electrodes also implemented for this reaction (e. g., copper, nickel, iron), which are all characterized by simultaneous NADRR and HER.[^21a^, ^22^] This is due to the low catalytic activity of carbon towards HER (similar was observed in mercury).^[24]^ In Figure 2b, NADRR in a flow cell on a flat GC electrode and porous carbon electrode on Toray paper support has been shown. Compared to the flat GC electrode, the porous carbon electrode has a much higher real surface area; this results in not only an increase in capacitive currents but also a shift of the NADRR onset toward less negative potentials. The experiments in Figure 2b have been conducted under stagnant conditions. As a consequence, instead of a well–defined limited current (Figure 2a) a peak has been observed in the forward direction and an apparent absence of a catalytic current for NADRR in the backward direction. These are further indications of mass transport limitations.

To investigate the distribution of NADRR products, the electrochemical potential was maintained at a constant level or periodically varied around a steady‐state value for an extended period (usually 1000 minutes). The composition of the products was initially assessed using HPLC and subsequently verified through Nuclear Magnetic Resonance (NMR) spectroscopy. Prior to delving into the discussion on how product distribution varies under different conditions, a comprehensive explanation of product quantification via HPLC is provided.

### Determination of Product Distribution

**Figure 3:**
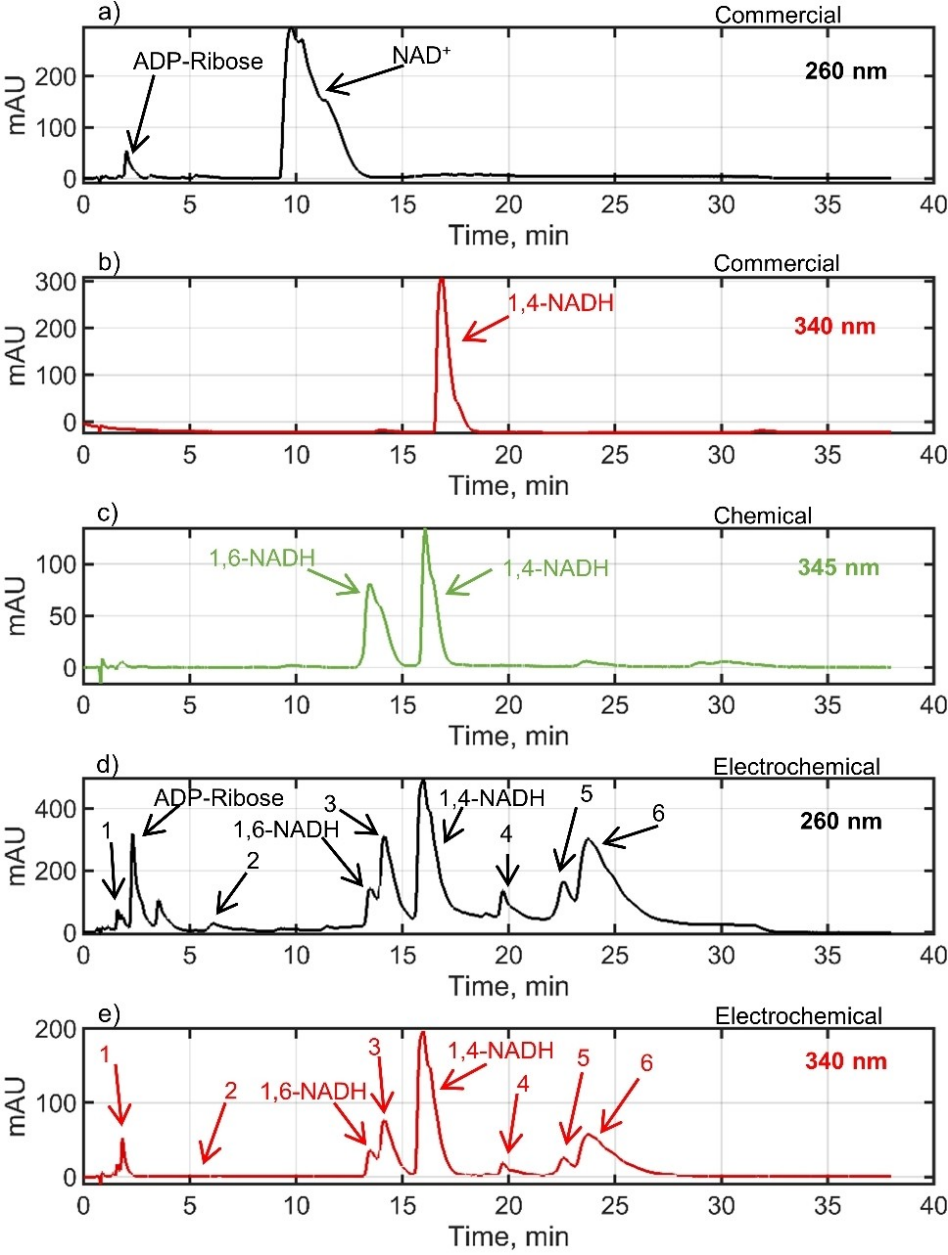
Comparative chromatographs of different samples: a) commercial sample of 500 μM NAD^+^ in 0.1 M phosphate buffer at 260 nm, b) commercial sample of 607.36 μM 1,4‐NADH in 0.1 M phosphate buffer at 340 nm, c) chemically prepared sample containing 1,4‐NADH and 1,6‐NADH at 345 nm (NaBH_4_ was used as a reducing agent with starting concentrations of NaBH_4_ and NAD^+^ in a 0.1 M potassium phosphate buffer (pH 6) of 70 mM and 1 mM, respectively), d) exemplary electrochemical sample at 260 nm and e) exemplary electrochemical sample at 340 nm. Each chromatograph displays the retention time on the x‐axis and the absorbance on the y‐axis, allowing for the identification and comparison of the peak patterns corresponding to the various compounds present in each sample. Notable peaks are labeled with their respective compound names or numbers for clarity. Electrochemical samples are collected after 19 h of NADRR at −1.082 V vs. Ag/AgCl on a porous carbon nanoparticle electrode on Toray paper support featuring 0.88 mg cm^−2^ carbon nanoparticle loading and 2.5 wt % PTFE in a flow cell setup where the starting concentration of NAD^+^ was 2.5 mM in 0.1 M sodium phosphate buffer (7.5 pH), at the flow rate of 80 ml/min and 22 °C.

All products of NADRR on unmodified electrodes[^13g^, ^14^, ^25^] show absorbance in the UV‐VIS spectra, with characteristic absorption maxima at 260 nm, for NAD^+^ & ADP‐Ribose, at 340 nm for 1,4‐NADH and different dimers (NAD_2_), at 345 nm for 1,6‐NADH and at 395 nm for 1,2‐NADH. All reduced forms (1,2‐NADH, 1,4‐NADH, 1,6‐NADH, and dimer forms) show also characteristic peaks at 260 nm. Due to the overlap of absorptions of different products at 260 nm and 340 (345) nm, it is difficult to obtain product distribution from UV‐VIS spectra. To overcome this, many groups used a combination of an enzymatic assay and UV‐VIS spectroscopy for the determination of product distribution.[^22^, ^26^] However, not all products can be quantified by this approach, and as discussed in the literature[^23a^, ^27^] enzymes are inhibited by NADH isomers (especially by 1,6‐NADH) as well as dimers.^[28]^ If the UV‐VIS determination is combined with product separation then individual product contributions can be separated. Chromatographs of different samples are shown in Figure 3.

**Figure 4:**
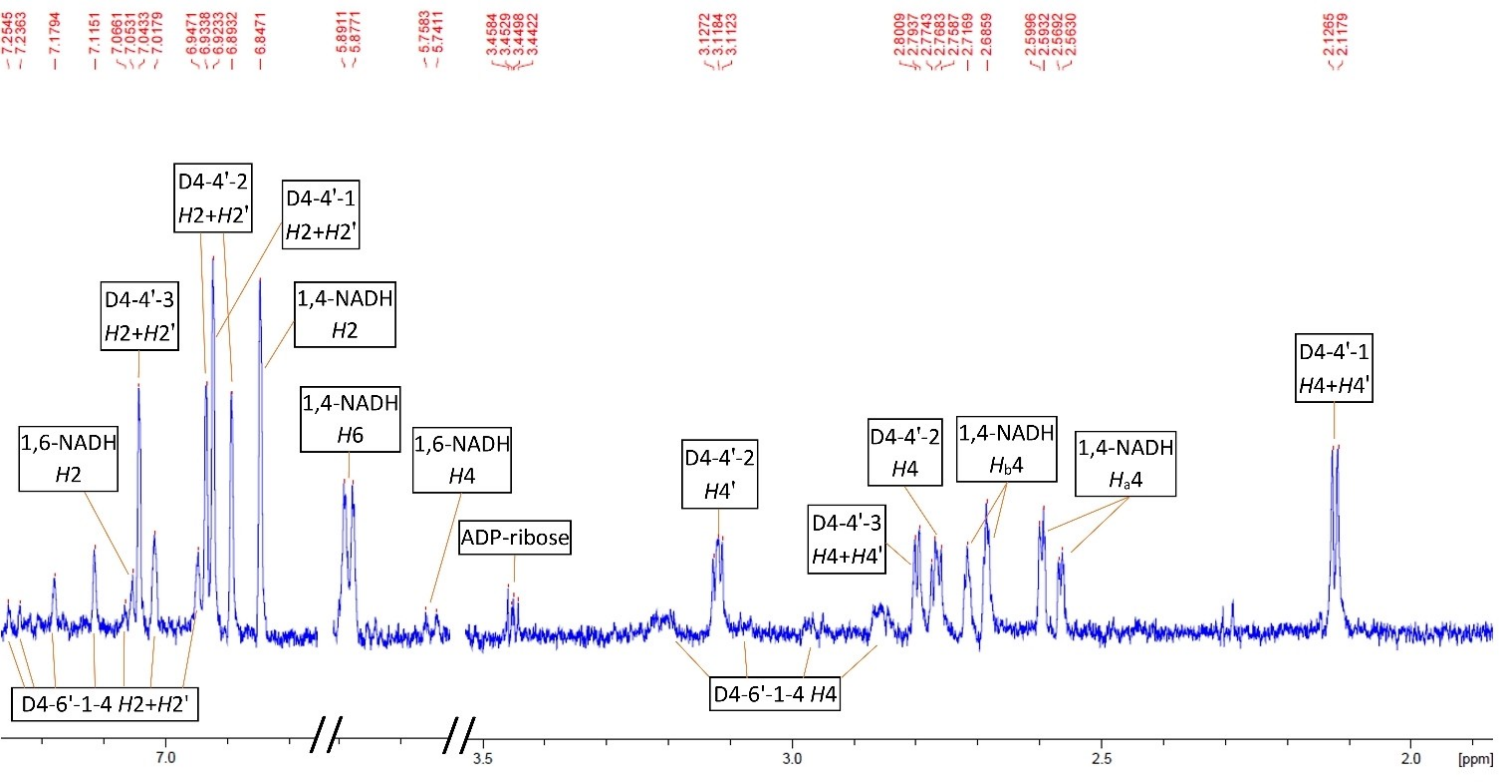
^1^H NMR spectra of the products of direct NADRR on a porous carbon nanoparticle electrode on Toray paper support featuring 0.88 mg cm^−2^ carbon nanoparticle loading and 2.5 wt % PTFE in a flow cell setup. Conditions of direct NADRR: initial NAD^+^ concentration 2.5 mM, steady–state potential −1.182 V vs. Ag/AgCl, 0.1 M phosphate buffer, 80 ml/min, sample taken after 1000 min.

As can be seen, a commercial sample of pure NAD^+^ (retention time ca. 10 min) contains also ADP‐Ribose (retention time ca. 2.3 min) as an impurity (Figure 3a). The commercial 1,4‐NADH sample shows a well–defined characteristic peak at 340 nm (Figure 3b). Chemical NAD^+^ reduction using NaBH_4_ as a reduction agent,^[29]^ resulted in the formation of two NADH isomers (1,4‐NADH and 1,6‐NADH) (Figure 3c). This aligns with the recent findings by Saba et al., which revealed that in a phosphate buffer at a pH of 7 or lower, only the 1,4‐NADH and 1,6‐NADH isomers persist in the reaction mixture.^[26]^ These authors proposed that the 1,2‐NADH isomer exhibits high instability and undergoes rapid decomposition under these conditions. Chromatographs of an exemplary electrochemical sample at two different wavelengths (similar to a chemical sample, no peaks were observed at a wavelength of 395 nm) are shown in Figures 3d&e. At 260 nm (Figure 3d), all products as well as non‐converted NAD^+^ exhibit characteristic peaks. In the present example, no peak for NAD^+^ was detected, indicating a complete conversion of NAD^+^ after 19 hours. Instead, a distinct peak for ADP–Ribose, a byproduct of NAD^+^ decomposition, was observed. At 340 nm, peaks corresponding to reduced forms of NAD^+^ appear (Figure 3e). The most prominent peak, observed at a retention time of approximately 16 minutes, was attributed to 1,4‐NADH (Figure 3e). At the characteristic retention time for 1,6‐NADH, both a shoulder and a peak were noted. Based on NMR results (Figure 4), the shoulder was tentatively identified as 1,6‐NADH. The peak at a retention time of 14.1 minutes was labeled as peak 3. Other minor peaks, observed at retention times of approximately 1.8, 6.3, 19.7, 22.5, and 23.9 minutes, were labeled as peaks 1, 2, and 4–6, respectively. The use of HPLC with UV‐VIS detection for analyzing the product distribution of NADRR on mercury was previously reported by Jaegfeldt.^[30]^ In that publication up to six dimers were observed at low overpotentials. At high overpotentials in addition to dimers, 1,4‐NADH and 1,6‐NADH were also detected. A recent NMR study^[22]^ has identified at least one dimer form on carbon electrodes. In order to clarify the products formed under the conditions of the present study, NMR analysis of the reaction mixtures after over 1000 min was carried out (Figure 4). The NMR analysis identified 1,4‐NADH and 1,6‐NADH forms under all conditions studied. In addition, ADP–ribose was identified in all samples. Furthermore, 7 different dimers were found, namely 3 isomers of 4,4′‐dimers, designated D_4,4’–i_, (i=1–3), and 4 isomers of 4–6′‐dimers, designated D_4,6’–i_, (i=1–4) (Figure 4). The 4–4’‐dimer isomers were dominant, while 4–6’‐dimers were present in trace amounts. The exemplary NMR spectra of these compounds are shown in Figure 4 (the NMR spectra for the sample corresponding to measurement at −1.082 V vs. Ag/AgCl (see other conditions in Table 1) can be found in SI (Figure S3). The agreement between the HPLC and NMR analyses is reasonable (Table 1). Similar results were reported by Jaegfeldt.^[30]^ This author also concluded that 4,4′‐dimers are predominantly formed, while 4,6′‐dimers are formed to a lesser extent. No 6,6′‐dimers were observed. No dimers of the form (2,4′ or 2,6′) were observed either. As discussed by Jaegfeldt,^[30]^ these forms should show characteristic peaks at 395 nm, which were not observed in the present study by HPLC. Therefore, we assigned peaks 2–6 in HPLC to dimers (Figure 3d&e). This correlates largely with previous reports (e. g., Jaegfeldt,^[30]^ Avigliano et al.).^[31]^ Peak 1 could also be a dimer as its concentration increases with time; its elution at very low retention times indicates a very polar isomeric form. It should also be noted that the distribution of the different isomers seems to be different from previous studies. This is related to the use of a different electrode material (here carbon, whereas in the literature mercury was used), which results in a different product distribution.

**Table 1:** HPLC and NMR analyses of product distributions from direct electrochemical NADRR using a porous carbon nanoparticle electrode on Toray paper support featuring 0.88 mg cm^−2^ carbon nanoparticle loading and 2.5 wt % PTFE in a flow cell setup at two distinct potentials, expressed as molar percentages of identified products. Conditions: 2.5 mM NAD^+^, 0.1 M phosphate buffer (pH 7.5), 22 °C, and 80 ml/min flow rate.

Method/ Species	HPLC		NMR	
	−1.082 V *vs*. Ag/AgCl	−1.182 V *vs*. Ag/AgCl	−1.082 V *vs*. Ag/AgCl	−1.182 V *vs*. Ag/AgCl
1,4‐NADH	38.2	33.1	33	31
1,6‐NADH	1.8	2.1	2	2
ADP–Ribose	11.2	14.2	4	4
ΣD	48.7	50.5	61	63

### Product Distribution under Electrochemical Conditions–Steady State Conditions

**Figure 5:**
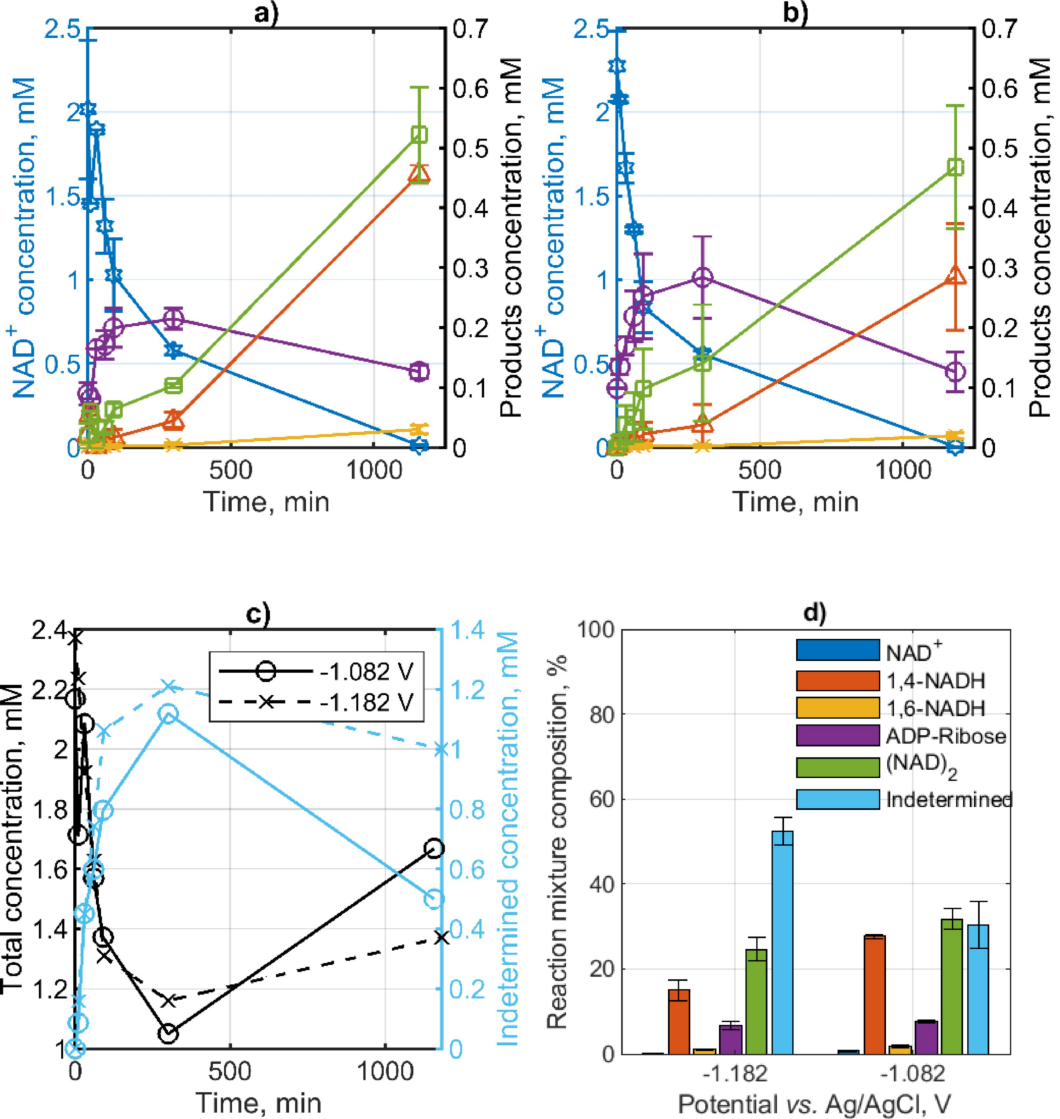
Products and reactant distribution over time of electrochemical NADRR at a constant potential of a) −1.082 V vs. Ag/AgCl and b) −1.182 V vs. Ag/AgCl c) calculated total concentration based on identified products and the calculated indetermined concentration and d) the composition of the reaction mixture at the end of the experiment at two different potential. Other conditions: porous carbon nanoparticle electrode on Toray paper support featuring 0.88 mg cm^−2^ carbon nanoparticle loading and 2.5 wt % PTFE was used in all measurements, 2.5 mM NAD^+^ 0.1 M phosphate buffer (7.5 pH), 22 °C, and 80 ml/min flow rate.

The impact of potential on the product distribution in electrochemical NADRR was investigated at two steady–state potentials, −1.082 and −1.182 V vs. Ag/AgCl, with an initial NAD^+^ concentration at approximately 2.5 mM. These potentials have been selected for two primary reasons: i) to minimize competition from the hydrogen evolution reaction, and ii) to maintain a low overpotential for NADRR. At both potentials, almost complete NAD^+^ conversion was achieved (Figure 5). The concentrations in Figure 5 are based on HPLC characterization of the reaction mixtures at different times. The molar absorption coefficients of NAD^+^, 1,4‐NADH, and ADP–ribose are based on the values given in Table S2. The molar absorption coefficient of 1,6‐NADH was not determined in the present study. The same value was used as for 1,4‐NADH, which may slightly overestimate the concentrations of 1,6‐NADH (Table S2, literature values). Dimer concentrations were calculated using an average of the molar absorption coefficients from Table S2. This literature value has been rescaled by a factor of 0.9 (based on the ratio between the HPLC 1,4‐NADH molar absorption coefficient determined in the present study and the 1,4‐NADH literature value reported by Jaegfeldt).^[30]^ ADP–ribose is present at the start of the experiment but it is also formed as a by‐product of NADRR. Similar was reported by Liu et al.^[22]^ As can be seen in Figures 5a & b, the concentration of ADP–Ribose is increasing and then decreasing with time. This may be due to further degradation to AMP, which is currently proven in our lab. The results indicate that 1,6‐NADH is formed in a very small amount at both conditions. Dimers are forming faster than 1,4‐NADH and represent the most dominant products at both conditions. To close the mass balance at different times, all identified products plus unreacted NAD^+^ were summed (Figure 5c). The results show that the mass balance is not closed at any time, but the deviations are larger at short reaction times. This can be taken as an indication of the formation of a reaction intermediate, which at longer reaction times converts into some of the detected products. The reaction mixture composition at two different potentials and after 1000 min has been shown in Figure 5d. Under both conditions, the largest part of NAD^+^ is converted into an unknown product (indetermined) and dimers (in sum ca. 49–63 %). The amount of 1,4‐NADH ranges from 18.6 % to 24.4 %, with an increased formation at −1.082 V vs. Ag/AgCl. The latter findings are consistent with those observed on copper electrodes^[15]^ and by Liu et al. on carbon–based electrodes.^[22]^ The amount of 1,4‐NADH is larger than that of 1,6‐NADH at all reaction times. This is contrary to the report by Liu et al.,^[22]^ where on carbon felt electrode 1,6‐NADH is present at a similar concentration as 1,4‐NADH. A possible reason for this discrepancy can be the longer reaction time (ca. 19 h) in the present case than in the literature study (ca. 1 h), but also different reactor configuration and flow conditions.

The product distribution based on HPLC was compared with the data obtained by NMR (Table 1). A reasonable agreement between HPLC and NMR data was observed. Note that indetermined products were not considered and only cumulative amounts of all dimers are shown (for dimer distribution see Table S3). It should be noted that the quantitative NMR data are less accurate than the HPLC data due to the measurement procedure. In particular, the ADP–ribose values are affected because the signal for ADP–ribose is so close to the water peak that it has by far the largest error of all. Also, the number of dimers identified by NMR is higher than the number of peaks observed by HPLC, indicating that some dimer isomers overlap in HPLC.

### Product Distribution under Electrochemical Conditions–Dynamic Conditions

**Figure 6:**
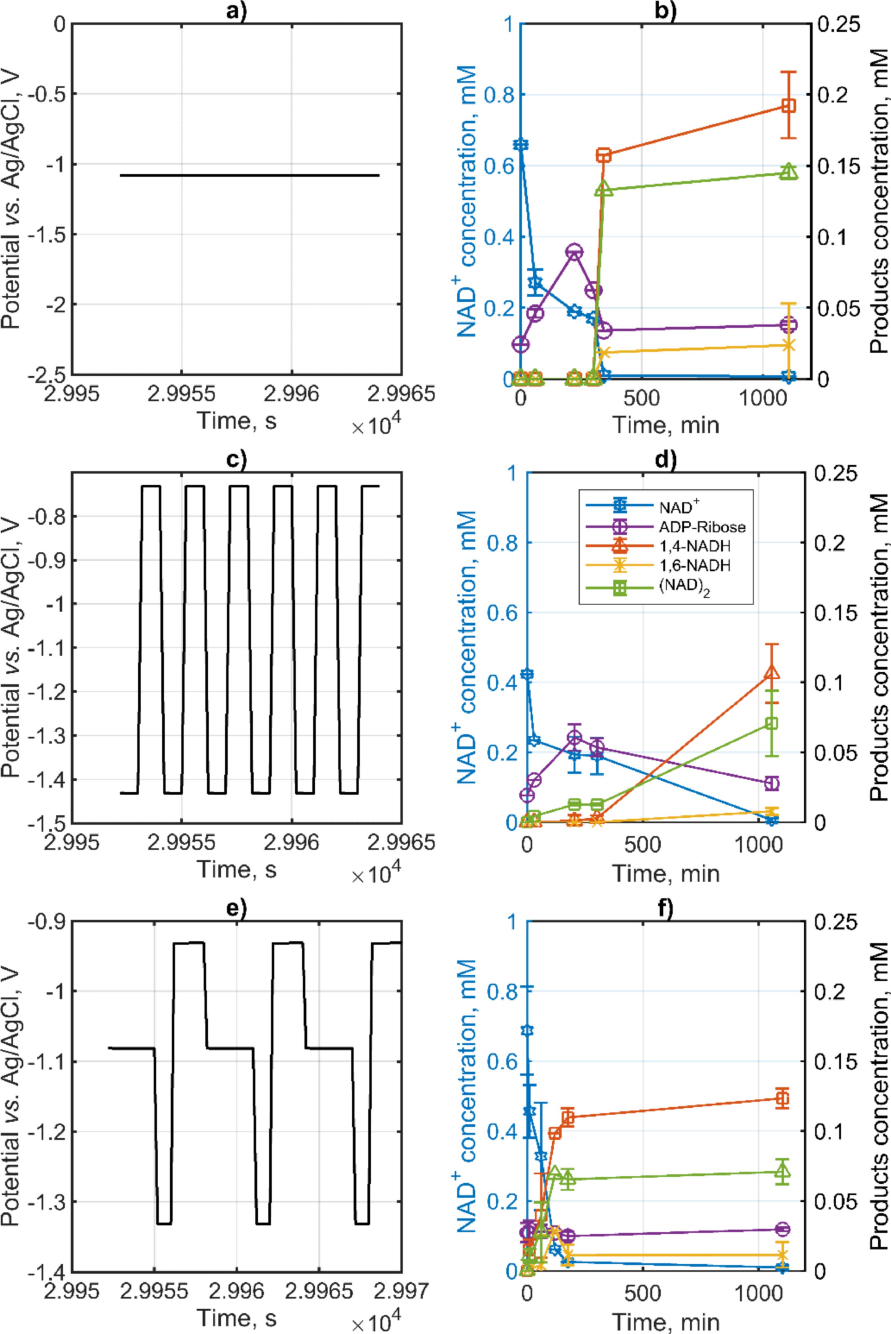
The influence of steady state and dynamic operations on product distribution of NADRR. The potential inputs a, c, & e) are of the steady state and dynamic operations (SS, DYN1, & DYN2) respectively. The reactant and product concentration changes are shown in b, d, & f) for SS, DYN1, and DYN2, respectively. Conditions: porous carbon nanoparticle electrode on Toray paper support featuring 0.88 mg cm^−2^ carbon nanoparticle loading and 2.5 wt % PTFE was used in all measurements, nominal NAD^+^ concentration 0.5 mM, dynamic inputs at the fundamental frequency of 0.5 Hz, for DYN1 and 0.16 Hz for DYN2. Other conditions 0.1 M phosphate buffer, flow rate 80 ml/min, and 22 °C.

The impact of dynamic conditions on the distribution of NADRR products was explored. For context, a steady–state experiment conducted at a potential of −1.08 V vs. Ag/AgCl served as a benchmark, aligning with the average potential values observed in two dynamic experiments (−1.08 V and −1.07 V vs. Ag/AgCl for DYN1 and DYN2, respectively). The comparison involved examining input profiles (either steady–state or dynamic) and the resulting product distributions under varying conditions, as depicted in Figure 6. Although the target NAD^+^ concentration for these experiments was 0.5 mM, actual measurements slightly varied from this figure. It was found that under none of the conditions was mass balance achieved, with the discrepancy in product accounting being most pronounced in the DYN2 experiment (0.11 mM, 0.22 mM, and 0.24 mM for SS, DYN1, and DYN2, respectively).

Generally, conditions featuring less negative overpotentials and lower concentrations yielded a more satisfactory mass balance closing. The hypothesis is that hydrogen generation at more negative overpotentials could trigger secondary reactions, leading to the formation of unidentified products. This is supported by an experiment tracking hydrogen production via GC over 19 hours, which showed a decrease in hydrogen quantity over time, as evidenced by Faradaic efficiency (Figure S1). In all scenarios, 1,4‐NADH was produced, with the highest concentration observed under steady–state conditions. Nonetheless, dynamic conditions, particularly DYN2, exhibited enhanced selectivity towards 1,4‐NADH at short reaction times but with the drawback of generating a significant volume of unidentified products. Moreover, while steady–state and DYN1 conditions exhibited delayed 1,4‐NADH formation kinetics, DYN2 conditions demonstrated rapid initial kinetics. In DYN2 experiments, product concentrations stabilized quickly and remained largely unchanged thereafter, hinting at a distinct mechanism of product formation under these conditions. These findings underscore the potential benefits of pulse operation in enhancing selectivity, though further research is needed to fine–tune pulse profiles, amplitude, and frequency to mitigate the identified drawbacks.

### Direct Electrochemical Co–Factor Regeneration Combined with Enzymatic Reaction

**Figure 7:**
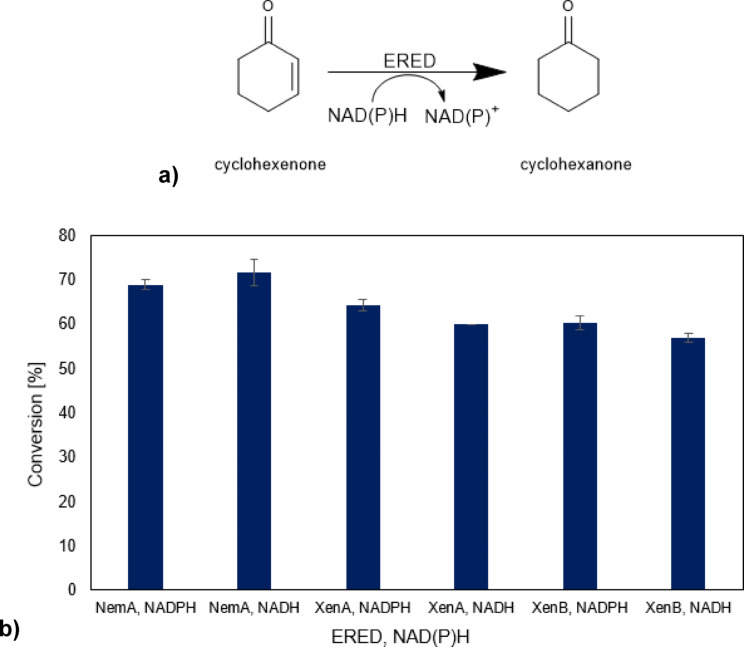
Substrate conversion from the EREDs NemA, XenA, and XenB from Pseudomonas putida. a) schematic representation of the enzyme–catalyzed reaction. b) substrate conversion with different cofactors (3 mM NADH or 3 mM NAD(P)H) and 3 mM cyclohexenone as starting material as determined after 4 hours of reaction time.

Finally, the validity and applicability of the direct electrochemical cofactor regeneration combined with the enzymatic cyclohexenone reduction into cyclohexanone, using an enoate reductase (ERED) from *Pseudomonas putida* (XenB) recombinantly expressed in *E. coli* and purified was investigated. Different EREDs were initially tested, with both 1,4‐NADH and 1,4‐NADPH cofactors (Figure 7b).

Although XenB indicated slightly lower product conversion, it was selected due to higher thermal stability; nano differential scanning fluorimetry (nanoDSF) measurements proved conformational changes at 30 °C for XenB, while for XenA the changes were noticed at 28 °C and for NemA at 25 °C. At the same time, it showed slightly higher specific activity against cyclohexenone with 1,4‐NADH as a cofactor, at 0.12 U/mg, compared to 0.10 U/mg for XenA and 0.11 U/mg for NemA. The apparent Michaelis–Menten kinetic parameters for XenB by varying the cyclohexanone concentration (0.05–10 mM) were determined to be *K*_m_=0.08 mM and *k*_cat_=0.02 s^−1^.

**Figure 8:**
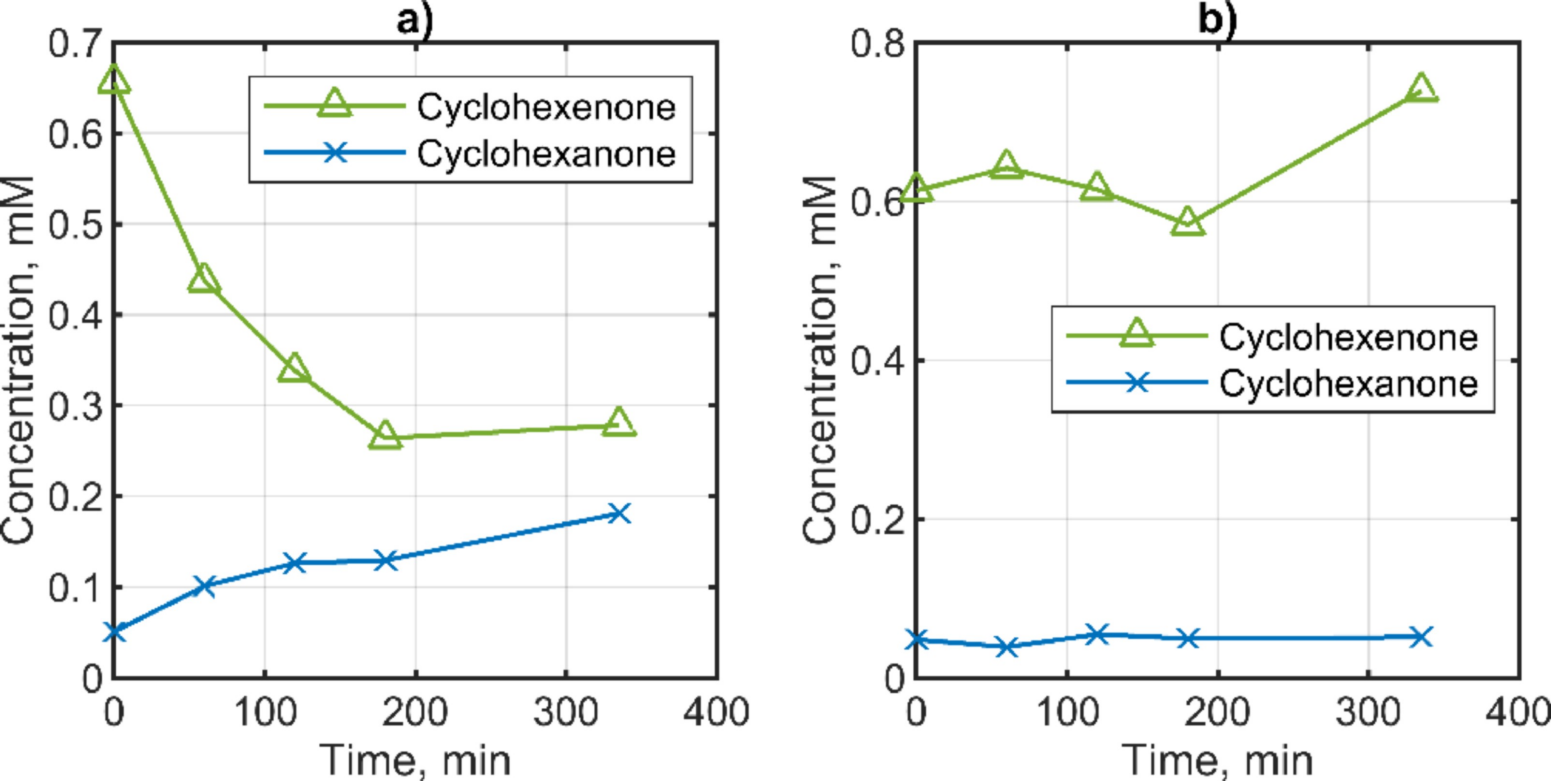
Conversion of cyclohexenone to cyclohexanone overtime during a) enzymatic reaction with XenB (0.17 mg/ml) in the presence of direct electrochemical 1,4‐NADH cofactor regeneration starting from nominal 0.5 mM NAD^+^ and b) control experiment in the absence of enzymes and cofactors. Conditions: direct NADRR has been performed under DYN2 conditions with a mean potential value of −1.07 V vs. Ag/AgC on porous carbon nanoparticle electrode on Toray paper support featuring 0.88 mg cm^−2^ carbon nanoparticle loading and 2.5 wt % PTFE in a flow cell setup; 0.5 mM for cyclohexanone (nominal concentration), 0.1 M sodium phosphate buffer, 7.5 pH, at room temperature (22 °C), 80 ml/min flow rate.

To demonstrate the efficacy of 1,4‐NADH produced in an electrochemical experiment when utilized with XenB, a reaction mixture was prepared by combining the products of electrochemical NADRR, obtained by applying a DYN1 potential input at a nominal NAD^+^ concentration of 2 mM, with 0.3 mg/ml XenB and 2 mM cyclohexanone. After 6 hours, a conversion rate of approximately 3 % was observed. This rate is notably lower than that achieved in previous experiments using commercially available 1,4‐NADH (Figure 7b). The primary reason for this reduced efficiency is the low (non‐stoichiometric) concentration of 1,4‐NADH, which was about 0.1 mM under DYN1 conditions (see Figure 6d). Furthermore, the presence of other components, such as 1,6‐NADH and dimers, in the electrochemical sample may have contributed to the inhibition of the enzyme‘s activity. As previously discussed, these components can act as enzyme inhibitors. In both cases, there was no in situ regeneration of 1,4‐NADH. In the next step, the XenB enzyme was added to an electrochemical reactor with a carbon‐based porous electrode in the presence of 0.5 mM cyclohexanone and 0.5 mM NAD^+^ at time zero. The electrochemical cofactor regeneration was conducted dynamically by applying DYN2 potential input (Figure 6e), and the product distribution over time was followed. In this case, only two components of the reaction mixture were monitored, namely cyclohexenone and cyclohexanone by using GC. The change of their concentrations over 5 h of reaction is depicted in Figure 8a. As can be seen, a decrease in the reactant concentration and an increase in the product concentration have been observed. It can be estimated that ~20 % of cyclohexenone is converted to cyclohexanone. In the control experiment without enzymes, concentrations of both cyclohexanone as well as cyclohexanone did not change over time (Figure 8b). Therefore, direct electrochemical regeneration of 1,4‐NADH in the presence of enzymatic reaction shows conversion to the product, but with lower conversion than in the biochemical experiment without electrochemical cofactor regeneration (Figure 7b).

The reasons for the lower conversion need to be further investigated. In the enzymatically catalyzed reduction of cyclohexenone with chemically generated 1,4‐NADH, which contained both enzymatically active and inactive isomers, the substrate product conversion was 10‐fold lower than in the reaction with 1,4‐NADH alone. In the chemically prepared sample, the amount of 1,6‐NADH isomer is very high compared to the electrochemical samples. The main products in the electrochemical samples were dimers. As reported in the literature, dimers also act as inhibitors (this was not tested for the enzyme used in this study).^[28]^ In addition, it is not clear whether undetermined products can affect enzyme activity.

## Conclusions

The influence of input potential pulsing on the product selectivity of direct NADRR on a carbon–based electrode has been investigated. Furthermore, product selectivity at two different NAD^+^ concentrations and steady–state overpotentials was shown. Liquid products of NADRR under different conditions were quantified by HPLC and NMR. 1,4‐NADH, various dimers, and ADP‐ribose were the major products identified under all conditions. The amount of 1,6‐NADH was low under all conditions studied. The mass balance was not completely fulfilled under all conditions studied. The deviation was more pronounced at higher concentrations and especially at higher overpotentials. Pulsing conditions improved the selectivity towards 1,4‐NADH compared to dimers. The product selectivity was very sensitive to the pulsing profile, indicating a possibility to further optimize the pulsing conditions. This is particularly important in view of the large amount of indetermined product(s) observed under pulsing conditions. Their chemical identity is currently unclear and requires further investigation. The possibility of bioelectrochemical synthesis combining enoate reductase and direct NADRR under pulsed conditions has been demonstrated. Future research directions may focus on mitigating enzyme inhibitory effects and optimizing conditions for efficient and selective cofactor regeneration in electroenzymatic processes. Overall, this work contributes to the developing field of electrochemical cofactor regeneration, bridging electrochemistry, and enzymatic catalysis for sustainable and selective synthetic strategies.

## Experimental Section

Two experimental setups were implemented: a rotating disc (RDE) (RRDE‐3 A, serial number M1716, produced by ALS), and a home–made flow reactor setup. In both setups, a reference electrode has been used to control/monitor the potential of a working electrode. The flow reactor system consists of a cathode (WE) and anode (CE) compartments (Figure 1). Both CE and WE were carbon‐based. The WE was connected to a reference electrode (RE) compartment. Both WE and CE compartments contained 35 ml of phosphate buffer solution which was circulated during experiments. The compartments were separated by a Nafion membrane (Nafion 117), which was placed in between two PTFE sealings. All compartments were made of polyether ether ketone (PEEK), except the endplates which were made of poly methyl 2‐methyl propanoate (Plexiglas). The working electrode in the RDE setup was a GC disc electrode with a diameter of 5 mm, while in the flow reactor, a GC or porous carbon nanoparticle electrode on Toray Paper (H23 T20 A, produced by Quintech) (further details are given in the SI) with an area of 4.1 cm×4.1 cm were employed. The Vulcan PF carbon nanoparticles with a particle size of 25 nm, produced by CABOT, and the PTFE binder (PTFE Dispersion DISP 30 (TFE‐DISP30, produced by Quintech)) were used. The counter electrodes were i) platinum mesh in the RDE and ii) a 4.1 cm×4.1 cm GC electrode in the flow reactor setups. The reference electrode was a reversible hydrogen electrode (RHE) in RDE− and Ag/AgCl (*E*=195 mV *vs*. RHE (25 °C)) in the flow reactor‐ setup. Before performing the electrochemical experiments, the GC electrodes were firstly polished with 0.3 μm alumina suspension and rinsed with Milli–Q water (IQ 7000), followed by polishing with a 0.05 μm alumina suspension and rinsing with Milli–Q water.

Sodium phosphate monobasic dihydrate, NaH_2_PO_4_ ⋅ 2H_2_O (≥99.0 %, 156.01 g/mol, Sigma–Aldrich), and disodium hydrogen phosphate dihydrate, Na_2_HPO_4_ ⋅ 2H_2_O (≥99.5 %, 177.99 g/mol, Merck) were used for buffer preparation. NAD^+^ (≥97.0 %, 663.43 g/mol, Thermo Fisher Scientific), and 1,4‐NADH (≥97.0 %, 709.41 g/mol, Carl Roth) were used in this study. Furthermore, cyclohexenone (≥98.0 %, 96.13 g/mol, Thermo Fisher Scientific), was implemented as a substrate of the enoate reductase.

Before the electrochemical experiments, the solution was purged with N_2_ (≥99.999 %, Linde), for 60 mins.

### Enzyme Production, Isolation, and Characterization

The enoate reductase XenB from *Pseudomonas putida* ^[11d]^ was used in this study. A single colony of *Escherichia coli* (*E. coli)* BL21 (DE3) harboring the gene encoding the enzyme on the pGASTON‐XenB plasmid was transferred into 4 ml lysogeny broth (LB) medium (25 g/L, Carl Roth) supplemented with ampicillin and was incubated with shaking for 16 h at 37 °C. Subsequently, 400 ml LB medium supplemented with the corresponding antibiotic was inoculated with 1 % (*ν/ν*) of the pre–culture. For cultivation, baffled Erlenmeyer flasks with a liquid–to–air ratio of 1 : 4 were used. The cultures were incubated at 37 °C with shaking (160 rpm) until an OD_600_=0.6–0.8 was reached. Protein production was induced with 0.2 % (*w/ν*) L‐rhamnose and performed by incubating at 25 °C, 160 rpm for 8 h for XenB. The cultures were harvested by centrifugation at 4500 g, 4 °C for 20 min. The cell pellet was resuspended in 100 mM NaPP (Na_2_HPO_4_/NaH_2_PO_4_) pH 7.5 which was followed by cell disruption at 1.379×10^7^ Pa (Maximator, Huber minichiller 300). The soluble fraction was separated from cell debris by centrifugation at 4500 g, 4 °C for 30 min.

The enzyme purification was performed using a Ni^2+^‐NTA column (ROTI®Garose–His/Ni NTA–Beads, Carl Roth) with a bed volume of 5 ml and gravity flow. The column was washed with dH_2_O, followed by 5 bed volumes of wash buffer (100 mM NaPP, 300 mM NaCl, 30 mM imidazole, pH 7.5). The supernatant was loaded onto the column after filtration (0.2 μm filters). The washing steps were carried out with five–column volumes of lysis buffer. The protein was eluted with elution buffer (100 mM NaPP, 300 mM NaCl, 300 mM imidazole, pH 7.5) and desalted on PD‐10 columns following the instructions of the supplier. The final protein solution was stored in 50 mM NaPP (pH 7.5) at 4 °C until further use. For the activity determination, 96‐well plates (UV transparent, Thermo Scientific) were used and all readings were done at 340 nm in replicates ( n≤3). The activity measurements were performed in a final volume of 200 μl of NaPP (pH 7.5), containing 1 mM of substrate, 0.3 mM NAD(P)H, and an appropriate amount of purified and desalted enzymes. The “blank” measurements were performed under the same conditions, without the enzyme. The NAD(P)H consumption was monitored at room temperature for 30 min.

For the quantification of the product conversion, reactions were set up in air–tight 2 ml glass vials (GC vials). The standard reaction mixture (1 ml) 3 mM cyclohexanone, from a 100 mM stock solution diluted in DMF, 3 mM NAD(P)H, and 7.7 μM desalted enzyme, in 50 mM NaPP pH=7.5, to a final reaction volume 300 μl.

### High–Performance Liquid Chromatography

To obtain the product distribution during electrochemical NADRR, a 1290 Infinity HPLC system (Agilent Technologies) was used. The device was equipped with a binary pump (Serial No. DEBAB00888), sampler (Serial No. DEBAP02995), thermostat (Serial No. DEBAK10714), and an Eclipse Plus C18 column with a pore size of 1.8 μm (PN 959758–902). Stepwise isocratic elution chromatography has been employed with the composition of the mobile phase changing stepwise. Mobile phase A was 100 % methanol, mobile phase B was 0.1 M phosphate buffer with a pH 6, and mobile phase C was 5 % methanol in water. Stepwise isocratic elution chromatography with mobile phases A and B was used as a pre‐conditioning procedure with blank samples, as well as for product distribution determination with real samples. A combination of mobile phases A and C was used as a post–conditioning procedure (wash method) to remove phosphate buffer crystals after each HPLC batch run. Further details on stepwise mobile phase profile changes over time are provided in the Supplementary Information (SI). The samples were kept at −30 °C before HPLC measurements while they were stored at 4 °C during HPLC measurements. The temperature of the column was kept at 15 °C during all HPLC measurements.

The HPLC was calibrated by using commercial NAD^+^, 1,4‐NADH, and ADP‐ribose samples. NAD^+^ and 1,4‐NADH calibration curves have been obtained by using 21 samples of NAD^+^ and 1,4‐NADH (further details are given in the SI), while 6 samples have been used to obtain the ADP‐Ribose calibration curve (further details are given in the SI). All calibration curves were linear in the whole range of studied concentrations with R‐square values of 0.9995, 0.9993, and 0.9991 for NAD^+^, 1,4‐NADH, and ADP‐Ribose respectively. For NAD^+^ and ADP‐ribose calibration signals at 260 nm were used, while for 1,4‐NADH calibration plots at both 260 nm and 340 nm were obtained.

### NMR–Spectroscopy

NMR spectra were recorded on a Buker AVANCE Neo 600 operating at 600.13 MHz for ^1^H. The qualitative 1D ^1^H NMR spectra were measured using a W5 water suppression sequence with gradients. The ^1^H NMR data were obtained over 64 scans with a 90° flip angle (90° pulse=12.14 μs), sweep width of 11.9 kHz, acquisition time of 2.74 s, relaxation delay of 1 s, and 64 k data points. The temperature for all experiments was held constant at 298 K. The 500 μl sample was solved in 250 μl D_2_O. The D_2_O was used as an internal lock. The chemical shifts were reported in ppm, downfield from 3‐(trimethylsilyl)propionic acid sodium salt (TMSP).

### Gas Chromatography

For GC analysis of the samples with cyclohexanone /cyclohexanone (further details are given in the SI), the GC‐FID 7890B device (Agilent Technologies), was used. The injection syringe of a maximum size of 5 μl was used to inject 2 μl of the previously extracted sample. Before each injection, the syringe was washed five times using a mixture of acetone and methanol. The parameters of the GC were set to 250 °C (temperature), 27137.07 Pa (pressure), 41 ml/min (total flow), 3 ml/min (septum purge flow), with a split mode with a ratio of 75 : 1 and a split flow of 37.5 ml/min. The parameters for the GC column (19091 N–133) were set to 0.5 ml/min (flow), 27137.07 Pa (pressure), 26.592 cm/s (average velocity), with a holdup time of 1.89 min. The post–run flow was set to 3 ml/min. The GC oven was set to 120 °C, with a 1 min equilibration time, and a post run temperature of 60 °C. During the analysis, the initial ramp was held for 12 minutes at 120 °C, followed by a 1.6‐min ramp to 200 °C with a rate of 50 °C/min. For detection, the FID front detector was used with the following parameters: 250 °C (heater), 400 ml/min (airflow), 30 ml/min (H_2_ fuel flow), 25 ml/min (makeup flow of nitrogen), with a helium carrier gas flow of 1 ml/min. The data rate for the FID front detector was 20 Hz, with a maximum peak width of 0.01 min.

## Conflict of interests

The authors declare no conflict of interest.

1

## Supporting information

As a service to our authors and readers, this journal provides supporting information supplied by the authors. Such materials are peer reviewed and may be re‐organized for online delivery, but are not copy‐edited or typeset. Technical support issues arising from supporting information (other than missing files) should be addressed to the authors.

Supporting Information

## Data Availability

The data that support the findings of this study are openly available in Edmond at https://doi.org/[10.17617/3.VVAJSV], reference number 12835.
