# Supplementary material for: Product Distribution of Steady–State and Pulsed Electrochemical Regeneration of 1,4‐NADH and Integration with Enzymatic Reaction
Source: ChemistryOpen. 2024 Apr 12;13(8):e202400064. doi: 10.1002/open.202400064 (PMC11319214; doi:10.1002/open.202400064)
Supplement: Supplementary file 1 — Supporting Information [file OPEN-13-e202400064-s001.pdf]

ChemistryOpen

Supporting Information

Product Distribution of Steady–State and Pulsed Electrochemical Regeneration of 1,4-NADH and Integration with Enzymatic Reaction

Mohammed Ali Saif Al-Shaibani, Thaleia Sakoleva, Luka A. Živković, Harry P. Austin, Mark Dörr, Liane Hilfert, Edgar Haak, Uwe T. Bornscheuer, and Tanja Vidaković-Koch*

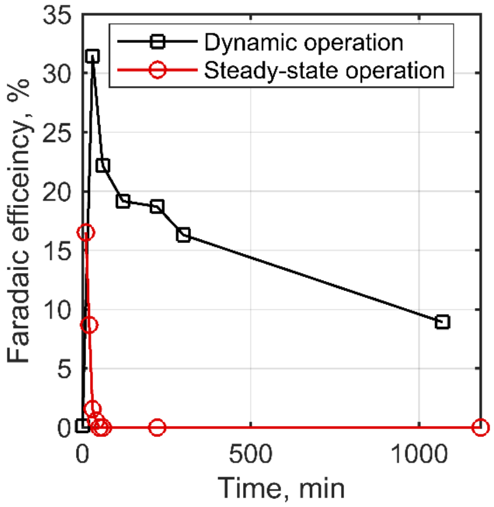

*Figure S1. The Faradaic efficiency for H_2 evolution in the flow-through reactor. The conditions are: porous carbon nanoparticle electrode on Torey paper support featuring 0.88 mg cm^{-2} carbon nanoparticle loading and 2.5 wt% PTFE, steady-state (SS) at -1.082 V vs Ag/AgCl, and dynamic operation (DYN1) at a mean potential value of -1.082 V vs Ag/AgCl, 0.5 Hz, and 80% amplitude. The reaction is performed with a nominal 0.5 mM NAD^+ concentration in 0.1 M sodium phosphate buffer, 7.5 pH, at room temperature (22 ^oC), at 80 ml/min flow rate, and 35 ml cathodic solution. In SS, the last three points are below the device determination level (**less than 1 ppm**). The hydrogen amount was measured with the help of an online gas chromatograph, Micro GC 3000 made by Agilent Technologies.*

Electrode preparation

The porous carbon electrode, measuring 41×41 mm², utilized in the experiments depicted in Figures 2- 6, and 8, was fabricated using the spray coating method on Toray paper (H23 T20A, produced by Quintech). The electrode, created using the ink specified in Table S1, featured a carbon loading of 0.88 mg/cm² and contained 2.5 wt% PTFE was employed in over 23 experiments, accumulating a total reaction time exceeding 278 hours. Initially, various tests were conducted to determine the optimal binder composition, as illustrated in Figure S2. Lowering the PTFE content is beneficial as it enhances the electrode's hydrophilicity. Therefore, increasing the PTFE content decreases the electrochemically active surface area.

Table S1 – The composition of the ink used for the electrode production

**Components**	**Mass (g)**
Carbon nanoparticles (catalyst)	1.22
PTFE (binder)	0.524
Water (solvent)	30

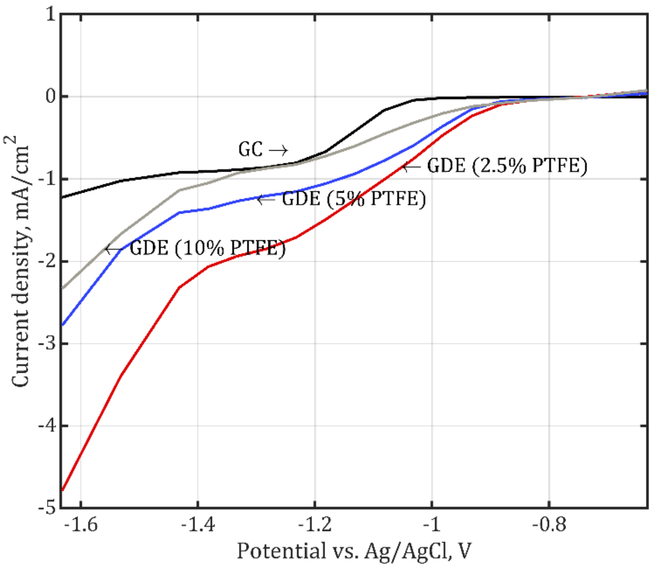

Figure S2. Influence of PTFE binder loading on NADRR. Porous carbon electrodes featuring ~0.7 mg cm^{-2} carbon loading on Toray Paper have been prepared with different PTFE wt % (2.5, 5, and 10 wt % (red, blue, and gray lines, respectively). For comparison NADRR on GC electrode in the RDE setup has been shown (black line). Other conditions are 50 mM sodium phosphate buffer, 2 mM NAD$^+$, 2000 rpm rotation rate. The experiments were performed with RHE as a reference electrode. All potentials were recalculated with respect to the Ag/AgCl reference electrode.

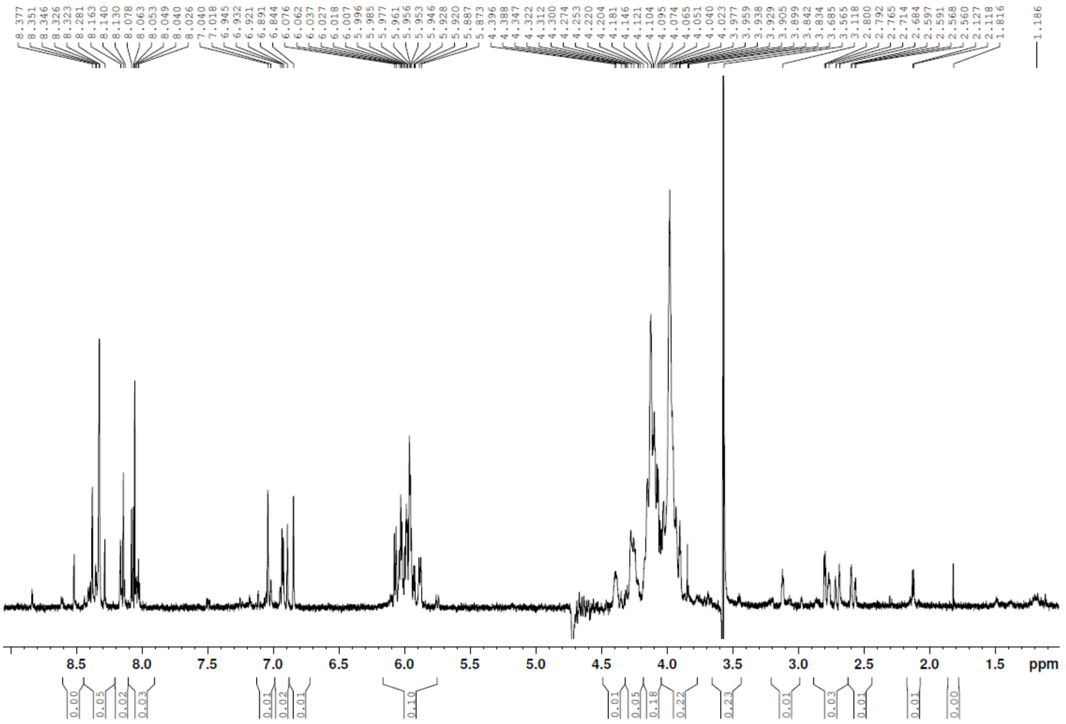

Figure S3. ^{1}HNMR spectra of the products of direct NADRR. Conditions of direct NADRR: porous carbon nanoparticle electrode on Torey paper support featuring 0.88 mg cm^{-2} carbon nanoparticle loading and 2.5 wt% PTFE was implemented, initial NAD$^+$ concentration 2.5 mM, steady state potential -1.082 V vs. Ag/AgCl, 0.1 M phosphate buffer, 80 ml/min, sample taken after 1000 min.

The molar absorption coefficients of NAD+, 1,4-NADH, and ADP-Ribose have been determined from the slopes of corresponding calibration plots (see Figures S4a,b & S5), based on the following equation[1]:

$$slope = \frac{A}{c} = \varepsilon \times l \times \frac{V}{F}$$

where *A* (mAU.s) is the integrated area of the peak, *c* (mM) is the concentration, ε (mM^{-1} cm^{-1}) is the molar absorption coefficient, *l* (cm) is the flow cell path length, *V* (ml) is the injection volume and *F* (ml/min) is the flow rate. The molar absorption coefficient values at different wavelengths have been summarized in Table S2. For comparison, the values determined by UV-VIS spectrometry, as well as values determined from the literature have been shown.

The HPLC values are a bit lower than UV-VIS values, but in general, they show a good agreement with both UV-VIS values (our lab) and literature values (a rescaling factor of ca. 0.9 compared to 1,4-NADH molar absorption coefficients determined in Jaegfeldt's lab [2] can be calculated).

Table S2– Molar absorption coefficients of different species determined by HPLC and UV-Vis methods

Method	HPLC		UV spectrophotometer		Literature	
Compound	ε_{260}, $mM^{-1}cm^{-1}$	ε_{340}, $mM^{-1}cm^{-1}$	ε_{260}, $mM^{-1}cm^{-1}$	ε_{340}, $mM^{-1}cm^{-1}$	ε_{260}, $mM^{-1}cm^{-1}$	ε_{340}, $mM^{-1}cm^{-1}$
ADP-Ribose	12.92	n.a.	n.d.	n.a.	13.5[3]	
NAD+	15.96	n.a.	17.1	n.a.	17.8[4]	
1,4-NADH	12.61	5.65	14.0	5.90	14.4[2]	5.15[5], 6.2[2]
1,6-NADH	n.d.	n.d.	n.d.	n.d.	21.30[2]	5.27[5], 6.5[2]
dimeric species (1-6)	n.d.	n.d.	n.d.	n.d.	30.20-38.20[2]	6.50-7.80[2]

Table S3– The comparison in terms of mole ratio for the HPLC and NMR analytics for two different potential -1.082 V and -1.182 V vs. Ag/AgCl. Conditions: 2.5 mM NAD+ 0.1M phosphate buffer (7.5 pH), 22 °C, and 80 ml/min flow rate.

Method	HPLC			NMR	
Species	-1.082 V *vs.* Ag/AgCl	-1.182 V *vs.* Ag/AgCl	Species	-1.082 V *vs.* Ag/AgCl	-1.182 V *vs.* Ag/AgCl
1,4-NADH	38.2	33.1	1,4-NADH	33	31
1,6-NADH	1.8	2.1	1,6-NADH	2	2
ADP-Ribose	11.2	14.2	ADP-Ribose	4	4
1	1.9	1.0	$D_{4,4'-1}$	**9**	**16**
2	0.4	0.2	$D_{4,4'-2}$	**22**	**21**
3	**14.1**	**18.8**	$D_{4,4'-3}$	**15**	**9**
4	2.7	3.7	$D_{4,6'-1}$	7	9
5	3.7	4.6	$D_{4,6'-2}$	3	2
6	**25.9**	**22.2**	$D_{4,6'-3}$	4	5
			$D_{4,6'-4}$	1	1
Σ1-6	**48.7**	**50.5**	ΣD	**61**	**63**

HPLC calibration tables and curves

Table S4 – Stepwise isocratic elution chromatography profiles for the HPLC NADRR product quantification, using 100% methanol as mobile phase A, and 0.1 M phosphate buffer with a pH 6, as mobile phase B. Flow rate of the mobile phase is 0.3 ml/min with a maximum pressure limit of 1050 bar, and post-run time of 2 minutes.

**Time, min**	0	10	15	20	22	30	31	38
**A, %**	1	1	5	5	5	5	1	1
**B, %**	99	99	95	95	95	95	99	99

Table S5 – Stepwise isocratic elution chromatography profiles for the wash procedure, using 100% methanol as mobile phase A, and 5% methanol in water, as mobile phase C. Flow rate of the mobile phase is 0.3ml/min with a maximum pressure limit of 950 bar, and post-run time of 2 minutes.

**Time, min**	0	10	15	20	22	28	35
**A, %**	1	1	5	5	5	1	1
**C, %**	99	99	95	95	95	99	99

Table S6 – Compositions of samples used for calibration with corresponding peak areas at 260 nm (NAD^+ and 1,4-NADH) and 340 nm (only 1,4-NADH).

**Concentration (μM)**		**Area NAD^+**	**Area 1,4-NADH**	**Area 1,4-NADH**
NAD^+	*1,4-NADH*	*260 nm*	*260 nm*	*340 nm*
2140.3	0	135500.8	0	0
0	2024.5	0	98813	44704
1926.3	81.0	121843.5	3904.7	1817.7
1712.2	202.5	108154.1	9765.8	4482.3
1605.2	303.7	101529.9	14903.6	6742.8
1498.2	404.9	96296.4	20037.1	8837.3
1391.2	506.1	89820.5	25050.1	11066.6
1284.2	607.4	81835	30620.6	13579
1177.2	708.6	76043.8	35045.8	15517.1
1070.1	809.8	68617.8	40670.7	18024.3
963.1	911.0	62349.9	45184	20133.7
856.1	1012.3	55325.8	51827.8	23022
749.1	1113.5	48593.7	57012.9	25356.4
642.1	1214.7	41749.4	62583	27839.8
535.1	1315.9	35361.9	67412.5	30048.9
428.1	1417.2	28441.6	72712.2	32460
321.0	1518.4	21199.5	77699.8	34752.8
214.0	1619.6	15449.4	81467.5	36631.2
85.6	1822.1	6434.4	92537.4	41656.5
10.7	5.1	708.6	253.6	118.7
5.4	10.1	360.1	702.9	313

Table S7 – The ADP-Ribose concentrations corresponding peak areas at 260 nm.

ADP- Ribose concentration (μM)	Area 260 nm
663.4	33981.7
331.7	17423.7
211.0	11363.9
105.5	5328.2
60.3	3616.8
28.3	1675.2

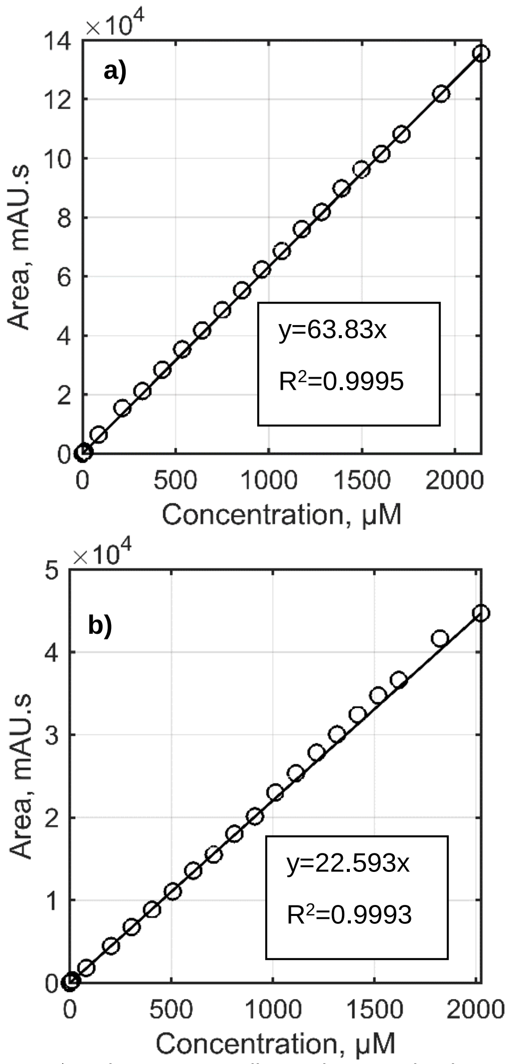

Figure S4 a and b. Calibration curves for NAD^+ and 1,4-NADH. All samples contained 100 mM sodium phosphate buffer, 7.5 pH.

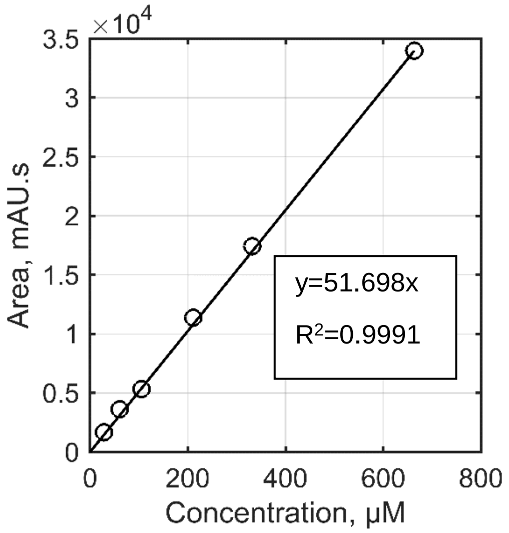

Figure S5. Calibration curve for ADP-Ribose. All samples contained 100 mM sodium phosphate buffer, 7.5 pH.

GC-FID calibration tables and curves

Preparing the internal standard

An internal standard was prepared by diluting acetophenone in ethyl-acetate, making 2 mM acetophenone solution (58.9 µl acetophenone in 250 ml volumetric flask). A special pipette (100-1000 µl) for volatile compounds for handling ethyl-acetate during preparation and extraction was used.

Preparation of the GC-FID samples

1. Put 600 µl of the sample into a 1.5 ml Eppendorf and add 600 µl of the internal standard. Always make sure to add the same amount of the internal standard as the experimental sample (for other volumes).
2. Vortex the Eppendorf with the sample for 30 seconds.
3. Centrifuge the Eppendorf for 2 min at 8500 rpm.
4. Collect up to 400 µl of the upper phase of the Eppendorf and place the 400 µl in a new Eppendorf.
5. Add anhydrous magnesium sulphate until the crystals are formed at the bottom and no magnesium sulphate particles are free-flowing in the Eppendorf.
6. Centrifuge the Eppendorf for 2 minutes at 8500 rpm.
7. Collect 220 µl from the upper phase of the Eppendorf and insert it into a GC-FID vial with an insert.
8. Keep the extracted sample in a tightly closed GC-FID vial with an insert in a 4°C fridge.

Table S8– Nominal and normalized cyclohexenone concentrations and corresponding peak areas used for calibration. Concentrations are normalized with respect to the internal standard (2 mM acetophenone in ethylacetate)

Cyclohexenone concentration (mM)	Area of cyclohexenone peak	$\frac{c_{cyclohexenone}}{c_{internal\ standard}}$	$\frac{A_{cyclohexenone}}{A_{internal\ standard}}$
0.500	2554272	0.250	0.112021298
0.400	1806600	0.200	0.074581452
0.375	1298359	0.1875	0.053946744
0.250	1223461	0.125	0.051867629
0.125	670341	0.0625	0.02744493
0.100	585605	0.050	0.023437273
0.050	282333	0.025	0.011812224

Table S9– Nominal and normalized cyclohexanone concentrations and corresponding peak areas used for calibration. Concentrations are normalized with respect to the internal standard (2 mM acetophenone in ethylacetate)

Cyclohexanone concentration (mM)	Area of cyclohexanone peak	$\frac{c_{cyclohexanone}}{c_{internal\ standard}}$	$\frac{A_{cyclohexanone}}{A_{internal\ standard}}$
0.450	3982024	0.225	0.166599587
0.400	3687855	0.200	0.147596529
0.375	3246984	0.1875	0.132937192
0.250	2016823	0.125	0.085501563
0.125	835304	0.0625	0.034706835
0.050	744383	0.025	0.030067046

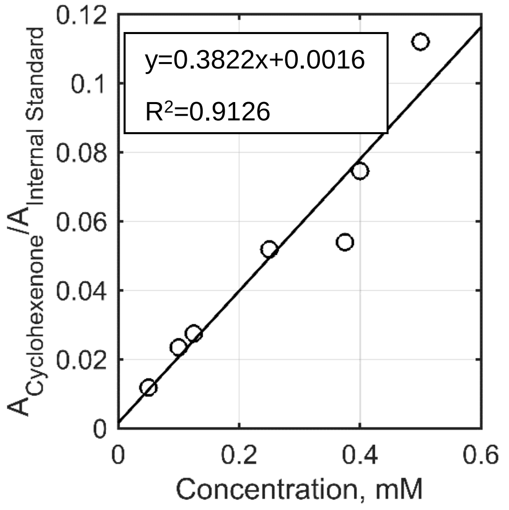

Figure S6. Calibration curve for cyclohexenone.

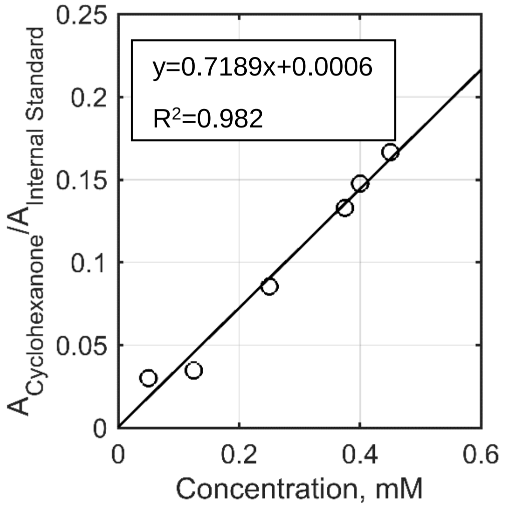

Figure S7. Calibration curve for cyclohexanone.

References

[1] C. Wang, S. Chen, J. Caceres-Cortes, R. Y.-C. Huang, A. A. Tymiak, Y. Zhang, *J. Chromatogr. A* **2016**, *1455*, 133–139.

[2] H. Jaegfeldt, *Bioelectrochem. Bioenerg.* **1981**, *8*, 355–370.

[3] H. L. Schultheisz, B. R. Szymczyna, J. R. Williamson, *J. Am. Chem. Soc.* **2009**, *131*, 14571.

[4] C. Bernofsky, S. Y. Wanda, *J. Biol. Chem.* **1982**, *257*, 6809–6817.

[5] T. Saba, J. W. H. Burnett, J. Li, P. N. Kechagiopoulos, X. Wang, *Chem. Commun.* **2020**, *56*, 1231-1234.
